# Sub-Ångstrom electric field measurements on a universal detector in a scanning transmission electron microscope

**DOI:** 10.1186/s40679-018-0059-4

**Published:** 2018-08-24

**Authors:** Jordan A. Hachtel, Juan Carlos Idrobo, Miaofang Chi

**Affiliations:** Center for Nanophase Materials Sciences, Oak Ridge National Laboratory, Oak Ridge, TN 37831 USA

**Keywords:** Differential phase contrast, 4D STEM, Sub-Ångstrom resolution, Octahedral tilts

## Abstract

Scanning transmission electron microscopy (STEM) excels in accessing atomic-scale structure and chemistry. Enhancing our ability to directly image the functionalities of local features in materials has become one of the most important topics in the future development of STEM. Recently, differential phase contrast (DPC) imaging has been utilized to map the internal electric and magnetic fields in materials from nanoscale features such as p–n junctions, skyrmions, and even from individual atoms. Here, we use an ultra-low noise SCMOS detector in as the diffraction plane camera to collect four-dimensional (4D) datasets. The high angular resolution, efficient high-SNR acquisition, and modifiability of the camera allow it to function as a universal detector, where STEM imaging configurations, such as DPC, bright field, annular bright field, and annular dark field can all be reconstructed from a single 4D dataset. By examining a distorted perovskite, DyScO_3_, which possesses projected lattice spacings as small as 0.83 Å, we demonstrate DPC spatial resolution almost reaching the information limit of a 100 keV electron beam. In addition, the perovskite has ordered O-coordinations with alternating octahedral tilts, which can be quantitatively measured with single degree accuracy by taking advantage of DPC’s sensitivity to light atoms. The results, acquired on a standard Ronchigram camera as opposed to a specialized DPC detector, open up new opportunities to understand and design functional materials and devices that involve lattice and charge coupling at nano- and atomic-scales.

## Background

Differential phase contrast (DPC) is an imaging mechanism used in scanning transmission electron microscopy (STEM) to produce an image that reflects the relatives shifts in the electron probe observed on the convergent beam electron diffraction (CBED) disks due to local electric and magnetic fields results [1, 2]. Although DPC was initially developed for the converged electron probe of the STEM, the technique has found significant success in X-ray and optical microscopy for measuring mesoscopic fields and biological samples [3–5]. More recently, advances in detector efficiency and electron probe aberration correction have brought DPC imaging back to STEM. In these latest results, researchers have used STEM-DPC to measure innate electric and magnetic fields of nanoscale phenomena such as p–n junctions [6], quantum wells [7], magnetic domains [8–10], ferroelectric polarizations [11], and skyrmions [12], even extending to mapping the fields surrounding individual atoms [13, 14].

Much of this new research has been brought on by advances in segmented detectors, and more recently in high-speed pixelated detectors. For segmented detectors, instead of using a standard circular or annular geometry, the detector is broken up into a series of adjacent segments that have been divided into quadrants [15]. A limited number of segments allow for fast efficient acquisitions and real-time atomic-resolution DPC imaging [13, 16, 17]. Alternatively, in the case of pixelated detectors, the detector segments are arranged into a Cartesian grid. With a pixelated detector, fully four-dimensional (4D) datasets can be acquired, where for each spatial position of the electron probe a two-dimensional (2D) image of the diffraction plane (a Ronchigram) is recorded [18–20]. The 4D datasets acquired with pixelated detectors can be used to measure the aberration-function of the probe [21], improve efficiency in DPC imaging [22], and more importantly, they can be used to retrieve and separate the phase of the electron probe and the sample via electron ptychography and holography [23–26]. Moreover, by acquiring the entire CBED pattern for each probe position, pixelated detectors inherently function as universal detectors because different types of STEM images [e.g., bright field (BF), annular bright field (ABF), and annular dark field (ADF)] can be reconstructed directly from a single 4D dataset [27]. While previous studies demonstrate the potential of probing internal electric fields in materials together with structure and chemistry at atomic resolution, the limit of spatial resolution accessible by DPC electric field mapping has not been evaluated. Achieving DPC with high spatial resolution is especially important to the studies of quantum materials where coupling behavior often occurs at an extremely small length scale.

Here, we demonstrate that electric field mapping through DPC imaging with sub-Ångstrom spatial resolution approaching the information limit of the microscope. The mapping is done by obtaining 4D diffraction datasets using a complementary metal–oxide–semiconductor (CMOS) camera. DyScO_3_ (DSO), a ternary lanthanide scandate, is used as a prototype material system in this work. The material is chosen because, when oriented in the [001] direction, the crystal exhibits alternating and ordered O-coordination and tilting on the heavier Sc and Dy sites. Furthermore, [001] DSO also holds a wide range of nearest-neighbor spacing in the projected plane, making it an ideal testbed for the spatial resolution of DPC imaging. The highly spatially resolved DPC field maps of DSO were used to determine the position of the light oxygen atoms and to analyze the azimuthal angles of octahedral tilts with single-digit angle accuracy. The results also show that this new generation of cameras can be used as universal detectors because they allow the reconstruction of BF, ABF, and ADF images from a single 4D dataset. It is important to emphasize that our experiments were performed using a fast, ultra-low-noise CMOS camera, which is more accessible in general microscopy laboratories than the specially designed segmented and pixelated detectors.

## Results and discussion

In a standard STEM acquisition, the electron probe raster across the sample, depending upon the interaction with the sample the electrons are scattered or diffracted out to be collected by different detectors at different collection angles to produce different images. For instance, the high angle annular dark field (HAADF) detector in the experimental setup used here covers collection angles of 80–200 mrad, where the high collection angles ensure the images are dominated by *Z*-contrast, meaning the intensity is dictated by the atomic number of the elements in the sample [28]. Alternatively one can use the ABF detector which collects the outer edge of Ronchigram, here the small collection angles (that neglect the inner part of the bright field disk) maximize the phase contrast transfer function enabling efficient imaging of light elements [29]. For each detector, each probe position is represented by a single scalar value corresponding to the number of counts on the detector during the dwell time at that probe position, creating a two-dimensional (2D) image during the scan.

**Fig. 1:**
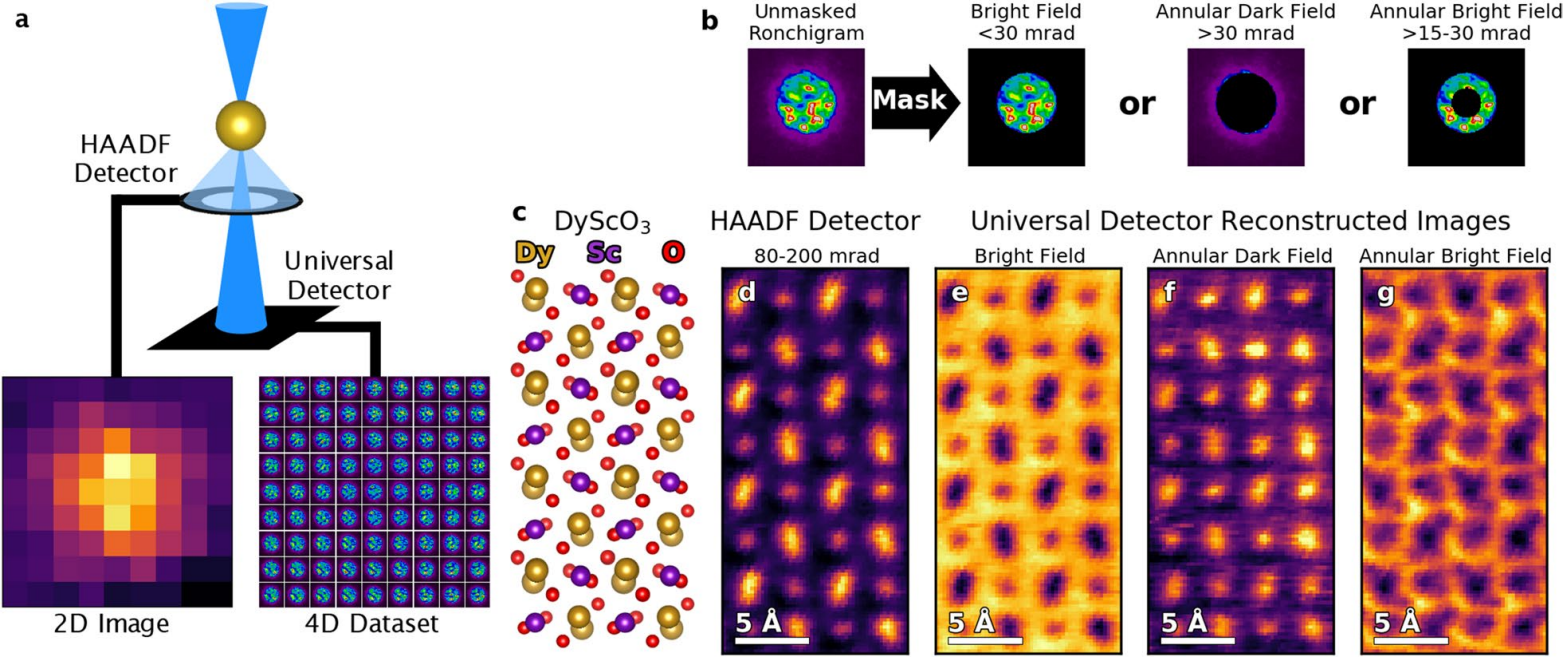
Four-dimensional data collection on a universal detector in a scanning transmission electron microscope. **a** Schematic of 4D-STEM acquisition. Standard detector: from 9 × 9 scan a 9 × 9 2D image is returned. Universal detector: from a 9 × 9 scan a 9 × 9 × 128 × 128 4D dataset is returned, where a Ronchigram is recorded for each probe position. **b** Different STEM signals can be reconstructed from a single 4D dataset by masking and integrating to the corresponding detector angles on each Ronchigram. **c** Crystal structure of DyScO_3_ (DSO). **d** STEM image acquired on standard HAADF detector. **e**–**g** Reconstructed STEM images from 4D dataset for **e** BF detector (Mask: < 30 mrad), **f** ADF detector (Mask: > 45 mrad), and **g** ABF detector (Mask: 15–30 mrad). The different reconstructed images have the same properties as images that would be recorded on dedicated detectors at those same collection angles

Conversely, with a pixelated detector the entire diffraction pattern is acquired for each probe position, resulting in 4D STEM datasets. The distinction between standard and pixelated detectors is shown schematically in Fig. 1a. For both types of acquisition, the electron probe rasters across an atom with a 9 × 9 scanning grid. From the HAADF detector, a 2D 9 × 9 *Z*-contrast image of an atomic column is produced, but from the universal detector a 4D 9 × 9 × 128 × 128 dataset where at each position in the 9 × 9 scanning grid a 128 × 128 pixel Ronchigram has been recorded providing resolution in both real-space and momentum-space. The 4D dataset can be used for advanced phase contrast analyses ptychographic measurements of the probe phase and modulus involving computationally intensive transformations and reconstructions [18, 30, 31]. However, there is also a wealth of information that can be extract directly from the 4D dataset, reducing the computational requirements and allowing for straightforward analyses.

Since the Ronchigrams that the universal detector records occur in the diffraction plane, the *x* and *y* positions of the pixels can be calibrated directly to collection angles, and the calibrated 4D dataset can be used to reconstruct the images that would have been acquired otherwise by genuine STEM detectors. As shown in Fig. 1b, this is achieved by masking each Ronchigram in the 4D dataset, and then integrating the unmasked signal. The probe used here has a convergence angle of 30 mrad, and to reconstruct a BF image we mask all pixels *outside* of a 30 mrad collection angle. For the ADF, we mask all pixels *inside* a 45 mrad collection angle, resulting in an integrated intensity of the reconstructed ADF image arising mostly from incoherent high-angle scattering, with a small contribution from the coherent BF signal. Additionally, an ABF detector can be produced by simply integrating only pixels between a collection angle of 15–30 mrad.

To demonstrate the image reconstructions, we perform a 4D dataset on [001] DSO, a material commonly used as a substrate for growing SrTiO_3_ with ferroelectric polarizations due to the strain induced from the DSO [32, 33]. The [001] DSO crystal structure is shown in Fig. 1c, and from this figure it can be seen that there are a wide range of interatomic spacings, ordered O-coordinations, and mixes of heavy and light atoms in close proximity. A HAADF image acquired on the actual HAADF detector of the [001] DSO lattice is shown in Fig. 1d, where the Sc atoms and the alternating tilts of the Dy-doublets can be distinguished. Figure 1e–g shows the universal detector reconstructions of different STEM signals corresponding to the Ronchigram masks from Fig. 1b. The reconstructed BF and ADF images both show the Sc and Dy-doublets with high spatial resolution. In addition, the *Z*-contrast, due to the relatively high atomic number of the Dy atoms with respect to the Sc atoms (*Z*_Dy_= 66, *Z*_Sc_= 21), is preserved in the ADF reconstructed image, even though the angular range is significantly reduced compared to the genuine HAADF detector, since the Ronchigrams collected range only extends to a semiangle ~ 60 mrad at the sides. Most importantly, in the reconstructed ABF image the O columns on either side of the Dy-doublets are clearly visible while they are unobservable in the as-acquired HAADF and the reconstructed BF and ADF images. The result demonstrates the effectiveness and versatility of 4D-STEM detector image reconstructions and that even the intrinsic aspects of the different STEM signals are faithfully reproduced in the universal detector.

**Fig. 2:**
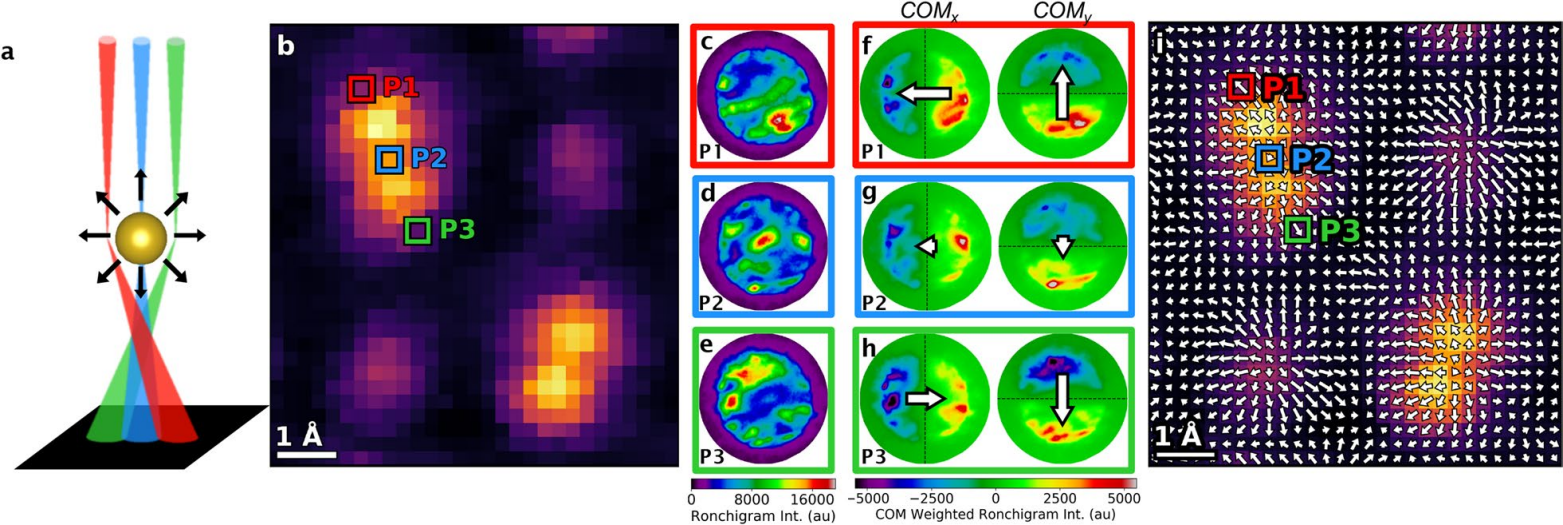
Measuring the electric field using DPC on a universal detector. **a** DPC generated by Coulomb attraction between negatively charged beam and positively charged nucleus causing deflection of the electron beam that is observable on a universal detector. **b** HAADF detector image of DSO unit cell with three pixels marked corresponding to acquisitions at three probe positions. **c**–**e** The Ronchigrams from the 4D dataset corresponding to the top-left (P1—**c**), midpoint (P2—**d**), and bottom-right (P3—**e**) of the tilted Dy double column. The deflection of the electron beam is quantified by weighting the intensity in each pixel of the Ronchigram by the distance of that pixel with respect to the center of the bright field disk in the *x*- and *y*-directions (with one half being positive and the other negative for each direction). The new weighted Ronchigram is then integrated. The resultant value is the *I*_COM_ value of the pixel which is proportional the electric field at that probe position. The COM-weighted Ronchigrams for the probe positions of P1–P3 are shown in **f**–**h**, respectively with the direction and magnitude of the I_CoM_ plotted as an arrow. (i) The direction and magnitudes of the field are plotted at each probe position in the across the entire unit cell

Beyond STEM image reconstructions, the combined angular and spatial resolution makes universal detectors sensitive to translational shifts in the CBED pattern, such as those that arise when the electron beam interacts with an electrostatic potential. For aberration-corrected electron probes that are small enough, the beam deflection induced by any electrostatic potential within the specimen can be detected. The effect is illustrated in Fig. 2a, which shows three probe positions relative to an individual atom. If the beam is outside of the electron cloud, the net field experienced by the beam from the atom is null. However, if the beam penetrates the electron cloud the force exerted by the highly localized positively charged nucleus outweighs the effect of the diffuse electron cloud and the electrons experience a radially inward Coulombic attraction as they pass near the nucleus. As a result, when the electron probe is positioned directly over the center of the atom, the electrons are influenced by the Coulomb force of the nucleus, but the force does not manifest itself as a deflection in the 2D projection plane. While when the electron probe is on the left side of the atom the average position of the probe electrons is shifted to the right, and when the probe is on the right side of the atom the position is shifted to the left.

The deflection of the beam can be quantified by calculating the shift of the center-of-mass (COM) of the diffraction pattern. Here, the mass in ‘center-of-mass’ refers to the position-averaged intensity maximum of the signal collected by the detector. Figure 2b shows a HAADF detector image of a single unit cell of [001] DSO with three pixels highlighted. One at the top-left of the Dy-doublet (P1—red), one right between the two Dy atoms (P2—blue), and one at the bottom-right of the doublet (P3—green). A 4D dataset is simultaneously acquired with the HAADF detector and we can examine the Ronchigrams corresponding to each pixel: P1—Fig. 2c, P2—Fig. 2d, P3—Fig. 2e. The shift in the COM due to the atomic field is clear in all three Ronchigrams; with the intensity from the top-left probe position concentrated in bottom-right of the bright field disk, the intensity from the midpoint probe position being generally symmetric, and the intensity from the bottom-right probe position being concentrated in the top-left.

The effect can be quantified for both the *x*- and *y*-directions by weighting the intensity of each pixel by its distance from the center of the BF disk in both the *x*- and *y*-directions [34]. The COM_X/Y_-weighted Ronchigrams (corresponding to Fig. 2c–e) are shown in Fig. 2f, g, h, respectively. For calculating COM_X_, the pixel positions on the left side of the Ronchigram are negative, while the right are positive, and for COM_Y_, the upper half pixels are negative while the bottom half are positive. Thus, when the entire COM-weighted Ronchigram is integrated it produces both a magnitude and direction for the COM shift that is proportional to the local electric field (annotated on the COM-weighted Ronchigrams with arrows) [23]. The process is repeated for each pixel in the 4D dataset, and the result is an atomic-resolution picture of the electrostatic field in the sample. As it can be observed in the figures, for the top-left of the Dy-doublet, the force that the electric field exerts on the probe points to the bottom-right (Fig. 2f), at the bottom-right of the doublet the force points to the top-left (Fig. 2h), and between the two Dy atomic columns there is very little deflection corresponding to a negligible field, and any measured direction and intensity is effective just noise (Fig. 2g).

The integration of the COM-weighted Ronchigram produce an amplitude and direction of the electric field with each probe position in the real space dimensions in the 4D dataset. The result is shown in Fig. 2i, where the magnitude and direction of the electric field force at P1, P2, and P3 can be observed alongside all of the neighboring pixels. The final electric field resolved figure shows the radially inward Coulombic attraction of the of the Dy and Sc nuclei on the negatively charged electron beam.

**Fig. 3:**
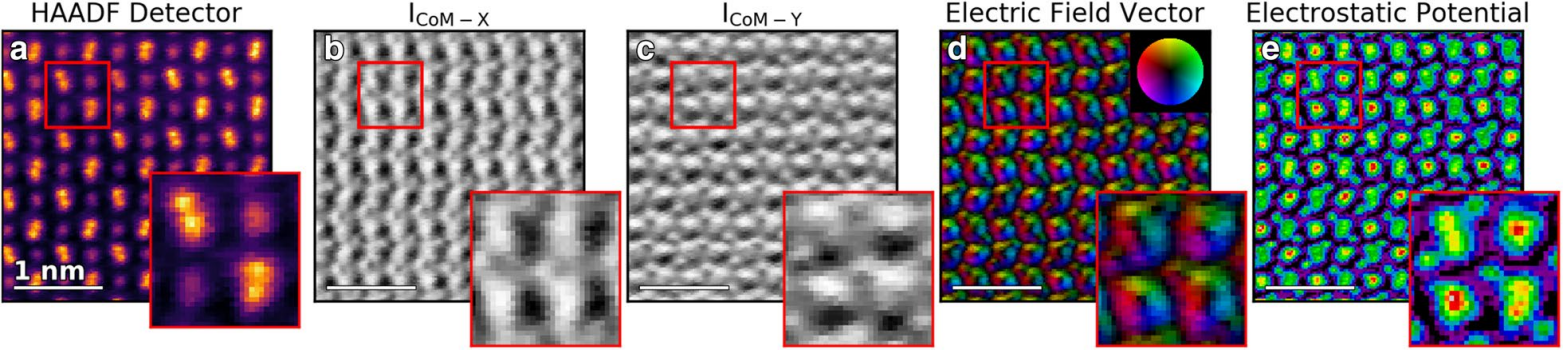
Atomic-resolution STEM-DPC imaging. **a** HAADF detector image of DySO, (inset) shows a zoomed view of a single unit cell. **b**, **c** *I*_CoM-X_ and *I*_CoM-Y_ images from the simultaneously acquired 4D dataset. **d** Direction-resolved DPC image where the color corresponds to the direction of the electric field, and the shade corresponds to the intensity. **e** Reconstructed atomic potential obtained through the inverse gradient method

It can also be seen in the areas on either side of the Dy-doublets (Fig. 2i) that intensity variations are present in the predicted vicinity of the O atomic columns. This result is better visualized through DPC imaging. Figure 3 shows the different DPC images extracted from the full 4D dataset (same dataset as the unit cell in Fig. 2). The image acquired simultaneously on the HAADF detector (128 × 128 pixels) is shown in Fig. 3a, with the unit cell from Fig. 2 in the inset. Figure 3b, c shows the *I*_COM-X/Y_ components, respectively, calculated from the COM_X/Y_-weighted Ronchigrams in the 4D dataset.

At each visible atomic site in the HAADF detector image there is a bright/dark spot at the same location in the *I*_COM-X/Y_ images, which is oriented left-to-right for *I*_COM-X_ and oriented top-to-bottom for *I*_COM-Y_. The change of contrast from bright to dark in the spots corresponds to the flip from right-to-left or bottom-to-top of the Coulomb deflection as the probe moves across the atomic column. In the insets of Fig. 3b, c it can clearly be seen that there are faint and small bright/dark spots where O atomic columns are present (known from the crystal structure shown in Fig. 1c, but which are not resolved in the HAADF image).

As shown in Fig. 2, the electric field magnitude and direction can be determined and imaged (Fig. 3d) by taking the *x*- and *y*-components of *I*_COM_ data and calculating the angle of the resultant electric field vector and plotting the direction as a function of color and the intensity as a function of shade (upper inset). From the field image, the characteristic annular shape of the in-plane projected electric field can clearly be seen for the heavy Sc and Dy atomic sites, and also quite clearly for the O-sites bordering the Dy-doublets. Furthermore, the resolution is sufficient to distinguish between the single column Sc atoms, where the inner annulus is circular, and the doublet column of the Dy sites where the inner annulus forms an elongated slot.

Additionally, using the inverse gradient method it is possible to reconstruct the atomic potential directly from the DPC-calculated electric fields [16]. Figure 3e shows the reconstructed atomic potential for the same 4D-dataset that produced Fig. 3a–d, here the O columns are clearly seen, and the separation of the two atomic columns in the Dy doublet can be observed.

The detection of the Dy-doublet via DPC is important as the spacing projected into the [001] direction of DSO is measured by X-ray diffraction to be 0.72 Å [35], which is approaching the 0.5 Å information transfer limit of the aberration-corrected microscope operated at 100 kV [36]. To test whether we have achieved this level of spatial resolution in DPC imaging, we perform post-acquisition interpolation to the raw data. The interpolation adds sub-pixel accuracy to the images. The as-acquired image has pixel size that is only 0.23 Å (128 × 128 pixel image with a field-of-view of 3 nm), which is nearly 1/3 significant fraction of the predicted interatomic spacing. In addition, to better distinguish between genuine electric field signatures and noise effects, local-low rank (LLR) denoising is performed to help with the identification of local minima and maxima [37].

**Fig. 4:**
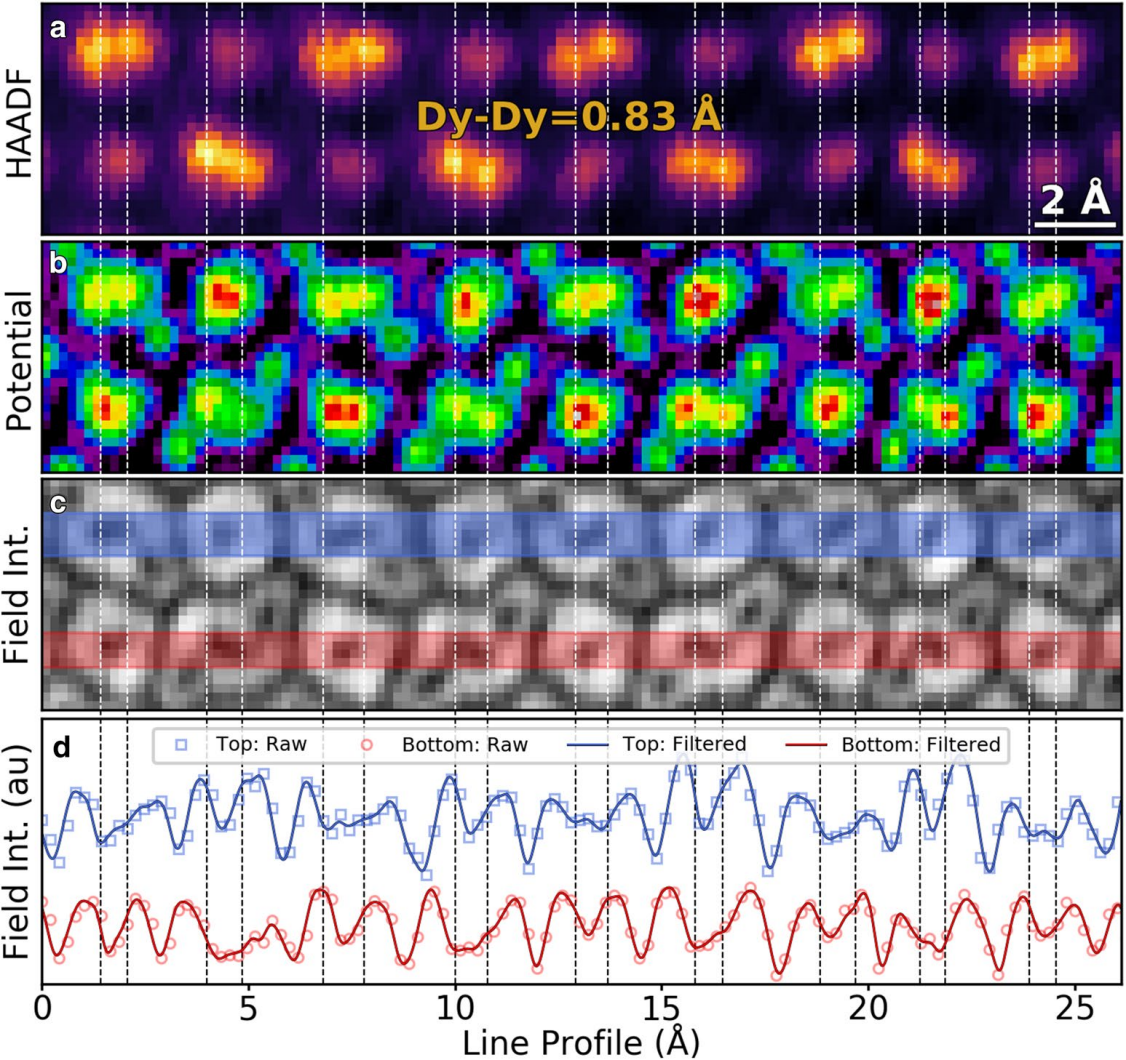
Sub-Å resolution in camera-based STEM-DPC. **a** HAADF detector image of DSO, showing the resolved Dy–Dy doublets in the [001] projection. Spacing measured as 0.83 ± 0.15 Å. Maps of **b** the atomic potential and **c** the electric field intensity from the simultaneously acquired 4D dataset. **d** Line profiles (width = 1 Å) of the electric field intensity map shown in **c** with a denoised and sub-pixel interpolated line profile overlaid. Vertical lines show the same regions in all four plots to demonstrate the doublet resolution. Data shown  here was obtained by cropping and rotating 90° Fig. 3a, d, respectively

The result of the LLR denoising and interpolation is shown in Fig. 4. The HAADF detector image (Fig. 4a) is used to identify the positions of the atomic Dy columns and measure the distance in a unit cell of DSO. From the HAADF images the positions of the Dy atoms can be measured directly. The Dy-doublets possess an average spacing as measured in the STEM of 0.83 Å ± 0.15 Å. This value is notably higher than the XRD spacing of 0.72 Å, but the difference is about half of the pixel size in the as-acquired 4D dataset and less than the standard deviation of the measured spacings. Thus, the STEM measurements are reasonably consistent with the values from X-ray diffraction, and still solidly within the sub-Å regime.

The post-processed atomic potential image from the simultaneously acquired 4D dataset is shown in Fig. 4b. A comparison between the HAADF image in Fig. 4a and the potential image in Fig. 4b demonstrates that the majority of the Dy doublets are clearly resolved by DPC. In Fig. 4c, the magnitude of the electric field of the same region is shown, where the two shaded regions correspond to the line intensity profiles plotted in Fig. 4d (blue: top region in Fig. 4c/top plot in Fig. 4d, red: bottom region in Fig. 4c/bottom plot in Fig. 4d). The two-line intensity profiles cover many Dy-doublets, so they are annotated on the figure with dashed lines (where the color of the dashed line corresponds to the color of the intensity profile, and the position is determined from the HAADF peak location). It can be seen here that the majority of the Dy-doublet sites are resolved in the electric field and atomic potential maps, as the line profiles exhibit distinct dips corresponding to the two columns in the Dy-doublets. We overlay the intensity profiles from the denoised image on the raw data from the same regions to demonstrate that the doublet resolution is present directly in the as-acquired dataset.

On most sites, the Dy-doublet minima in the field amplitude image correspond well with their the maxima in HAADF and the atomic potential, however it can be observed from the annotations that in some cases there is an offset, likely due to the relatively large pixel sizes used during acquisition (0.23 Å) and the distinction in the way scalars and vectors sum (since the potential and HAADF sum as scalars, but the field sums as a vector). Hence, a local minimum in the electric field magnitude may not always correspond to an atomic column, the same way a local maximum does in HAADF or a DPC measurement of the atomic potential. However, even when the local minima in the DPC do not directly correspond to the maxima in HAADF, the two dips corresponding to the doublet are still clearly observed in the majority of the Dy sites, indicating that the doublet is resolved in DPC field measurements. The ability to discriminate between the two Dy sites, in both field and potential, demonstrates that deep sub-Å resolution can be achieved in camera-based DPC and not just on high-speed segmented and specialized pixelated detectors.

The higher sensitivity toward light atoms in crystal lattices allow DPC to detect the O columns next to the Dy sites that are undetected by HAADF (as seen in Figs. 3 and 4). While the O atoms neighboring the Dy-doublets are observable, the O atoms bordering the Sc columns (shown in Fig. 1c) are not independently resolved. However, by examining the atomic potential maps in Figs. 3 and 4 it can be seen that the profile of the potential surrounding the Sc atoms is elongated along an axis. Moreover, the elongation axis is tilted and the tilt alternates between layers, indicating that the elongation is due to the two unobserved O columns in the DSO lattice.

The atomic potential of the O atoms combines with the field from the nearby Sc and Dy atoms. For the Dy site O columns, the spacing between the O and Dy sites is 1.77 Å, which is far enough for there to be a true local maximum at each O site. While the Sc site O columns only have a spacing of 0.73 Å meaning the Sc atomic potential is still significant at the location of the O atom preventing it from being imaged directly. To directly determine the positions of the Sc–O atoms requires comparison with simulation to de-convolve the signals from the Sc and O atoms. However, the location of the O atoms along the azimuthal tilt axis of each Sc atom extends the potential profile in that direction. Thus, while the exact position of the O columns cannot be directly determined from the DPC image, the influence of the field of the O columns is still resolved.

**Fig. 5:**
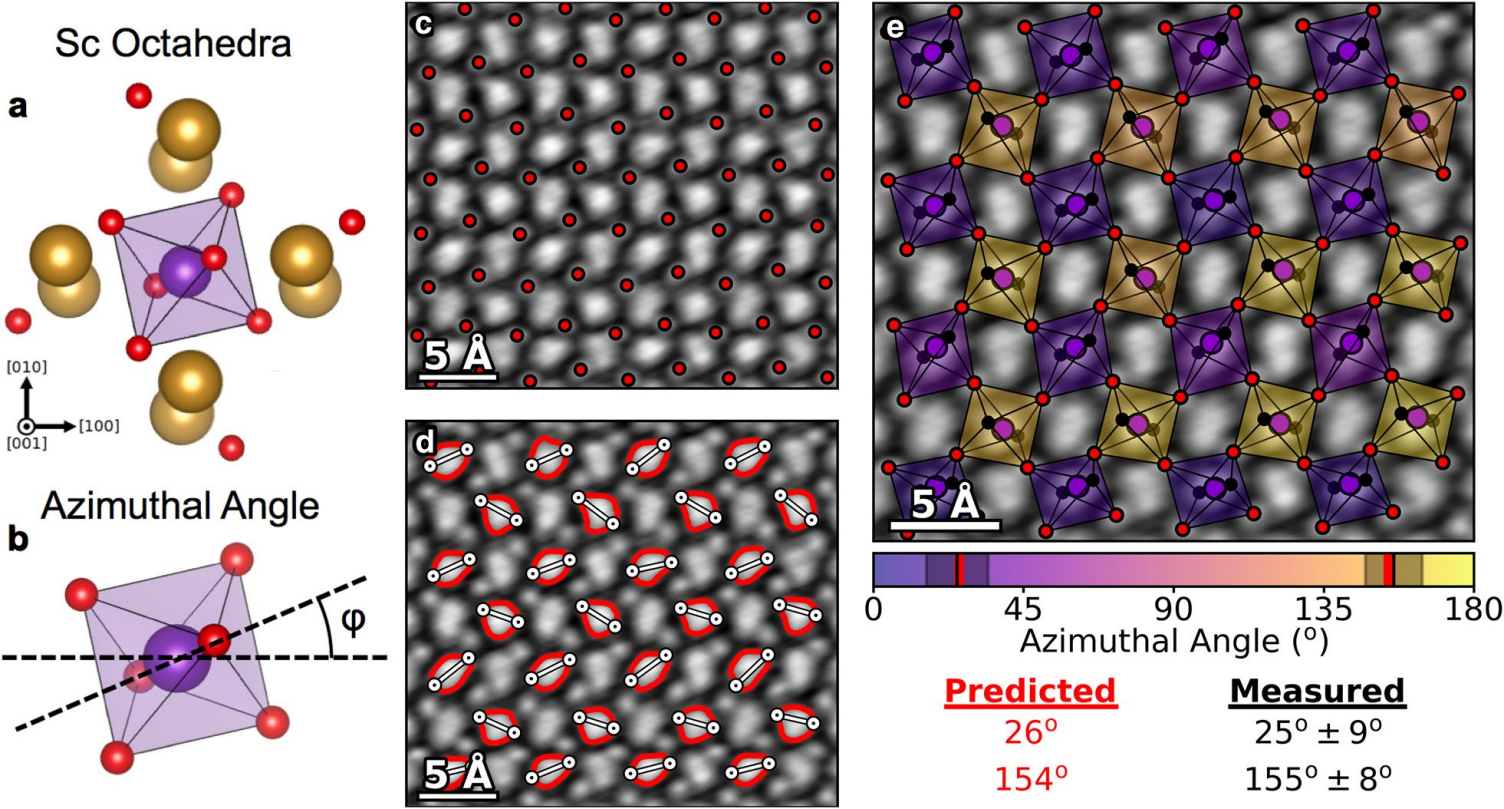
Measuring and mapping octahedral tilts with DPC. **a** The O-coordination of a Sc atom in DSO. **b** Azimuthal angle of octahedral tilt (polar angle not measured). **c** In-plane O atomic columns of the Sc octahedral found by identifying the local maxima near the Dy-doublet sites. **d** The azimuthal angle measured by taking isoline contours of the atomic potential from around each Sc site and identifying the long-axis of the elongated annulus. **e** Mapped Sc octahedrals where the color corresponds to the azimuthal angle of the octahedral tilt, demonstrating high accuracy of the measurements via STEM-DPC

The elongation of the DPC potential profile allows for additional analysis to be performed on the DSO structures, since the Sc atoms in DSO possess an octahedral coordination. Figure 5a shows an Sc atom and the surrounding Dy-doublets and O atoms. It can be seen that the four nearest-neighbor O atoms (the ones resolved near the Dy-doublets) form the in-plane O atoms of the Sc octahedral, and that the two unresolved O atomic columns form the upper and lower vertices in the out-of-plane direction. Since these out-of-plane vertices are unresolved, they cannot be plotted directly. However, the elongation caused in the DPC image does correspond to the azimuthal angle of the octahedral tilt, as shown in Fig. 5b.

One can visualize and plot the octahedra in the DSO lattice, by finding the positions of the in-plane O atoms for each Sc octahedra directly from identifying the local maxima in the DPC atomic potential image (Fig. 5c). To measure the azimuthal angles, we establish isoline contours of the DPC potential surrounding each Sc column. Then, we use an iterative algorithm to find the two positions on the isoline that are furthest from one another to establish the long-axis of the elongated DPC field annulus. The isoline contours and long-axis extrema for each Sc site are plotted in Fig. 5d.

The Sc octahedra are plotted and overlaid on the atomic potential map in Fig. 5e, where the shade and color of the octahedron sides corresponds to the measured azimuthal angle of the octahedral tilt. Note that since the out-of-plane O atomic columns are not directly identified from the data, the final two vertices are placed using the predicted structure and the measured azimuthal angle. Since the position is not measured the vertices are plotted as solid black dots as opposed to the red circles corresponding to the in-plane O atom columns.

The plot in Fig. 5e reproduces the alternating azimuthal tilt of the Sc octahedra from the predicted structure extremely well. For an ideal crystal, the azimuthal angles of the alternating tilts should be 26° and 154°, the measured values are 25° ±  9° and 155° ±  8°, matching the predicted values from X-ray diffraction to within a degree. The predicted azimuthal tilt angles are marked by a red line on the colorbar, and the averages and standard deviations of the measured angles are marked by black lines and shaded regions, respectively, to demonstrate the range of measured angles obtained through this method. The strong match demonstrates that highly accurate measurement of the octahedral tilts can be obtained from DPC.

## Conclusions

In conclusion, we have demonstrated sub-Å measurements of electric fields and electrostatic potentials in materials through 4D-STEM using a fast, ultra-low-noise CMOS camera, which is much more accessible than the specialized pixelated or segmented detectors previously reported. In addition, we have shown the sensitivity of camera-based DPC to subtle changes in light elements allowing for the mapping of oxygen octahedral tilts in perovskites, therefore allowing simultaneous analysis of charge distribution and lattice distortion quantitatively at sub-Å resolution. The coupling between charge and lattice is known to be the key factor dictating functionalities in materials, especially quantum materials [38, 39], demonstrating the value of 4D STEM based DPC to the discovery of new materials for information science and technology. Most of the research on atomic-resolution DPC has just been released in the last 2–3 years, and the newest generation of experiments are pushing the limitations of what can be achieved, and what effects can be observed [40, 41]. The information-limit resolution DPC measurements shown here with high accuracy and precision on a universal detector are now generally available through easily installed cMOS cameras on a STEM. Opening up the possibility for DPC to become a standard form of STEM imaging that all microscopists and materials scientists can routinely include in their suite of characterization tools.

## Methods

### STEM acquisition parameters

All data are acquired on a Nion UltraSTEM™ 100 operated at an accelerating voltage of 100 kV [36]. The probe current is 20 pA with a convergence angle of 30 mrad. The universal detector is a Nion 2020 Ronchigram camera, with a Hamamatsu ORCA ultra-low noise scientific CMOS sensor with a 2048 × 2048 pixel display. The camera can operate at a rate of 400 megapixels per second, with a read-out noise of 1.6 well *e*^−^ (RMS). Such low read-out noises allow for a DQE > 0.5 over wide range of exposure times, which is ideal for 4D STEM where the normally long acquisition times cause instabilities to compromise the spatial resolution of the image. The camera is not a specialized 4D STEM detector and is straightforwardly equipped to any STEM in place of the existing Ronchigram camera. For the datasets shown in this manuscript the display is cropped to a 256 × 256 pixel region and then binned to read out 128 × 128 pixel Ronchigrams at 760 frames per second. This places it between dedicated pixelated detectors, such as the JEOL pnCCD, which can readout a 256 × 256 pixel region at 2000 frames per second, and direct electron detectors which can readout 256 × 256 pixels at ~ 400 frames per second. For the dataset shown in Fig. 1 the acquisition time was 6 ms per Ronchigram image, while the data in Figs. 2, 3, 4, and 5 were acquired at 3 ms per Ronchigram. All acquisitions were performed over a 3 nm field-of-view. The pixel size and field-of-view were chosen to minimize drift and charging during image acquisition. The camera length of the microscope was adjusted to produce a diffraction plane calibration of 1 mrad per pixel.

The defocus condition provides a pivotal role in the detected DPC signal. With segmented detectors, the DPC signal can be read out live allowing for precise tuning of the defocus condition, however with our detector this is not currently possible. To achieve high quality DPC datasets we restrict ourselves to extremely thin regions of crystalline materials < 10 nm where the ideal DPC defocus conditions align more closely to the ideal HAADF defocus conditions and tune the defocus from the HAADF signal. Additionally, we perform small data acquisitions on unit cell sized regions that can be processed quickly to verify that the defocus conditions are sufficient for DPC before acquiring large datasets. For the atomic potential images additional high-pass filtering is used to remove long-range contrast, and enhance the signal originating from the atomic nuclei.

### Denoising and interpolation

The denoising scheme used in this manuscript is based in an LLR algorithm which has been implemented using principal component analysis (PCA). For details of the algorithm implementation see Ref. [37]. The LLR is applied only on the reconstructed 2D DPC images, not on the CBEDs themselves. The PCA tool used was the RandomizedPCA function available in the sci-kit learn Python library. The 2D interpolation tool used to provide sub-pixel accuracy in the analysis of the DPC images was the interp2d function available in the SciPy library.

### Finding atomic column positions

To identify the position of atoms in the datasets, we used the peak_local_max function in the sci-kit learn Python library. The position of the Dy and Sc atoms are determined from HAADF detector image, while the O atom positions are determined from the atomic potential image. The peak_local_max function does not have sub-pixel accuracy, hence the accuracy of the found atomic positions is only precise to within the pixel size (0.23 Å).

### Measuring the azimuthal angle

The azimuthal angle is determined by taking the isoline contours of the atomic potential. It is found that a fractional intensity of 0.45 is the highest intensity which results in the Sc contours being separated from the surrounding contours of the Dy and O columns. The extrema of the long-axis are determined iteratively by taking the point on the contours that are furthest to left and right (smallest and largest *x*-coordinate). The point on the contour furthest from each initial extremum is determined to find new left and right extrema, and the new and old positions are averaged. The process is repeated from the averaged positions until the new and old extrema positions converge to the same point.

## Abbreviations

STEM: scanning transmission electron microscope
DPC: differential phase contrast
4D: four-dimensional
CBED: convergent beam electron diffraction
2D: two-dimensional
BF: bright field
ABF: annular bright field
ADF: annular dark field
CMO: complementary metal–oxide–semiconductor
DSO: DyScO_3_
HAADF: high-angle annular dark field
COM: center-of-mass
LLR: local-low rank
PCA: principal component analysis
4D: four-dimensional
